# Influence of macronutrient composition of commercial diets on circulating leptin and adiponectin concentrations in overweight dogs

**DOI:** 10.1111/jpn.13285

**Published:** 2019-12-26

**Authors:** Niels Roderick Blees, Jeannette Wolfswinkel, Hans Sjoerd Kooistra, Ronald Jan Corbee

**Affiliations:** ^1^ Department of Clinical Sciences of Companion Animals Faculty of Veterinary Medicine Utrecht University Utrecht The Netherlands

**Keywords:** adipokine, adiponectin, dogs, leptin, macronutrients, overweight

## Abstract

Leptin and adiponectin play important roles in obesity‐related inflammation and comorbidities. Previous research suggests that alterations in dietary macronutrient composition can modify circulating leptin and adiponectin concentrations in people, but limited research on this subject has been performed in dogs. This study investigated the effects of commercial high protein (HP), high fat (HF) and high carbohydrate medium protein (HCMP) diets on baseline (T_−1_) concentrations, post‐prandial peak concentrations and total release in a ten‐hour time span of leptin and adiponectin in dogs, when compared to a maintenance high carbohydrate low protein (HCLP) diet. Thirty‐six overweight dogs were fed the HCLP diet in a one‐week control period, after which the animals were assigned to one of three groups. In three four‐week periods, each group was fed all test diets in a different sequence. At the last day of each period, blood was sampled at one hour before feeding (T_−1_) and at three (T_3_), six (T_6_) and nine (T_9_) hours after feeding. Feeding caused peak leptin concentrations at T_6_ and T_9_ (*p* < .001). No significant post‐prandial change in adiponectin concentrations was found (*p* = .056). The HP diet resulted in lower leptin peak concentrations (*p* = .004) and AUC_T−1–T9_ (*p* = .01), but none of the diets influenced baseline leptin concentrations (*p* = .273). Baseline adiponectin concentrations were lower for the HF diet (*p* = .018) and HCMP (*p* < .001), and the HP, HF and HCMP AUC_T−1–T9_ (*p* < .001) were lower compared with the HCLP diet. Female dogs had lower adiponectin baseline concentrations (*p* = .041) and AUC_T−1–T9_ (*p* = .023) than male dogs. In conclusion, the HP diet was associated with the lowest post‐prandial peak leptin concentration and the least decrease in adiponectin release, suggesting that a HP diet may improve immune‐metabolic health and post‐prandial satiety in overweight dogs.

## 1 INTRODUCTION

In dogs, overweight and obesity are common disorders that shorten the lifespan and cause severe comorbidities, such as osteoarthritis and insulin resistance (German et al., 2009; Kealy et al., 2000, 2002). These are often caused by altered release of adipokines, bioactive substances produced by white adipose tissue (Bastien, Patil, & Satyaraj, 2014; Radin, Sharkey, & Holycross, 2009).

Adipokines have physiologic functions in energy homeostasis and immune response regulation, via balanced release of inflammatory and anti‐inflammatory factors (German et al., 2009; Radin et al., 2009). In overweight conditions, hypertrophy and hyperplasia of adipose tissue have detrimental effects on this balance. Studies in overweight dogs showed increased release of inflammatory cytokines and decreased release of anti‐inflammatory adipokines in comparison with lean dogs (Bastien et al., 2014; Park, Lee, Oh, Seo, & Song, 2014). It is therefore implied that canine obesity is associated with continuous low‐grade inflammation, which in turn contributes to the development of comorbidities (Bastien et al., 2014; German et al., 2009).

Leptin has pro‐inflammatory and immune‐modulating actions, and an increase in fat mass results in increased circulating leptin concentrations, indicating an important role in signalling energy status (Cortese, Terrazzano, & Pelagalli, 2019; Orr & Davy, 2005; Radin et al., 2009). Leptin additionally induces a feeling of satiety after feeding and promotes energy expenditure through fatty acid oxidation and sensitising effects on peripheral insulin receptors (Minokoshi et al., 2002; Orr & Davy, 2005; Radin et al., 2009). However, persistent high leptin concentrations, associated with leptin resistance, promote inflammation and development of comorbidities, and could lead to a lack of satiety after feeding with increased difficulty to induce weight loss (Cortese et al., 2019; Radin et al., 2009). In addition, loss of leptin‐induced insulin‐sensitising effects assists in the development of insulin resistance and hyperinsulinemia (Jeusette et al., 2005).

Adiponectin, on the other hand, is released from adipocytes during periods of fasting to increase food intake and reduce energy expenditure (Lee & Shao, 2014). Adiponectin increases insulin sensitivity and has an anti‐inflammatory effect, which counteracts insulin resistance and low‐grade inflammation. Studies in overweight dogs show a reduction in adiponectin release, probably due to inhibition of gene expression by pro‐inflammatory cytokines (Park et al., 2014; Radin et al., 2009).

Several studies have described improvement of immune‐metabolic health after weight loss in overweight dogs (Bastien et al., 2014; German et al., 2009). However, it is often hard for owners to uphold energy restriction and weight loss is not always achieved (German, Holden, Bissot, Hacket, & Biourge, 2007). In humans, a high‐protein diet in combination with exercise and calorie restriction decreased leptin and increased adiponectin concentrations, thus improving insulin sensitivity and lowering inflammation (Ata et al., 2010). Another study in obese and diabetic humans used a low carbohydrate or a low fat, calorie‐restricted diet, and decreased circulating leptin and increased circulating adiponectin concentrations (Vetter et al., 2010). Altogether, these studies suggest that diet may play an important role in modulating adipokine release.

Information on the effects of macronutrient composition of diets on leptin and adiponectin concentrations in dogs is limited. One study did not find an effect of protein content of diets on baseline leptin concentrations when combined with neutering (Kawauchi et al., 2017). Another study combined diet with weight loss and revealed lower leptin concentrations with diets containing starches with a low glycemic index and higher adiponectin concentrations in diets with added diacylglycerols, when compared to diets with added triacylglycerols (Mitsuhashi et al., 2010). This study aimed to find out whether commercially available high protein, high fat or high carbohydrate diets can modulate baseline and post‐prandial concentrations of leptin and adiponectin in overweight dogs.

## 2 MATERIALS AND METHODS

### 2.1 Animals

As overweight dogs have different adipokine levels when compared to lean dogs (Park et al., 2014), 36 mostly overweight, but otherwise healthy experimental Beagle dogs (body condition score (BCS) range 5‐8/9 and age range 1–12 years), housed in the research kennel of the Faculty of Veterinary Medicine at Utrecht University, were included (Table 1). Dogs had 3–4 hr of voluntary exercise per day and had voluntary outdoor access. All dogs were intact, and at the trial start, three dogs had a BCS of 5/9. The other dogs had become overweight due to previous excess feeding of a maintenance diet, before the start of the trial. Body weight (kg) and BCS assessment and physical examinations were performed to ensure the health status of each dog prior to the start of the trial. Body condition was determined using a 9‐point scale, as validated by Laflamme (Laflamme, 1997). Estimations of the daily energy requirement (DER) were made using the energy intake, BCS and weight of each dog prior to the study. The protocol and study design were approved by the Animal Ethics Committee at Utrecht University (registered under number AVD1080020184847) and the Royal Canin Ethics Committee.

**Table 1:** Group characteristics of all three groups and changes in body weight and body condition score (BCS) at the end of the dietary trial

Characteristics	Group		
	Group 1	Group 2	Group 3
Number of dogs	12	12	12
Gender			
Male	4	4	4
Female	8	8	8
Age (years)	4.4 ± 3.2	6.3 ± 2.8	4.5 ± 3.6
Median BCS (range)			
Start trial	6 (6–7)	6 (5–8)	6.5 (5–7)
End trial	6 (6–7)	6 (5–7)*	6 (5–7)
Body weight (kg)			
Start trial	13.3 ± 2.2	12.2 ± 2.0	13.0 ± 2.0
End Trial	13.5 ± 2.3	11.9 ± 1.8**	12.9 ± 2.1
Sequence of diets after maintenance diet	HF, HCMP, HP	HCMP, HP, HF	HP, HF, HCMP

Note

Values expressed as mean ± *SD*, unless stated otherwise.

Abbreviations: BCS, Body condition score; HCMP, High carbohydrate medium protein diet; HF, High‐fat diet; HP, High‐protein diet.

* Significant difference (*p* < .05) compared with trial start by the Wilcoxon signed‐rank test.

** Significant difference (*p* < .05) compared with trial start by paired *t* test.

### 2.2 Trial design and management

To avoid confounding by gender, stratified randomisation based on gender was used to assign each dog in one of three groups (Table 1). After a control period of one week, in which a high in carbohydrates and low in protein (HCLP) maintenance diet was fed, the groups were fed one of the following dry diets: (a) a high‐protein diet (HP); (b) a high‐fat diet (HF); and (c) a high carbohydrate and medium protein diet (HCMP), when compared to the maintenance diet (Table 2). After a four‐week period, the diets were changed without run‐in period, with all groups having the three diets in a different sequence (Table 1). At the last day of each four‐week period, 2 ml blood was collected at one hour before feeding after an overnight fast (T_−1_) and at three (T_3_), six (T_6_) and nine hours (T_9_) after feeding to determine baseline concentrations and post‐prandial kinetics for both leptin and adiponectin, the latter consisting of the post‐prandial peak concentrations and the total release via area under the curve calculations for ten‐hour release. Each diet was fed isocaloric to avoid changes in body weight and body condition. Portion size of each diet was based on DER estimations and was adjusted to keep the animals overweight. The animals were fed once a day in the morning with ad libitum availability of water. All dogs ate their food within 30 min, and food bowls were removed one hour after feeding.

**Table 2:** Main ingredients and composition of each diet as stated by the manufacturer

	Maintenance diet	High‐fat diet†	High carbohydrate, medium protein diet†	High‐protein diet†
Brand name	Hill's Science Plan Canine Adult Advanced Fitness‐ Lamb & Rice	Royal Canin Gastrointestinal	Royal Canin Gastrointestinal Low Fat	Royal Canin Diabetic
Main protein source	Lamb meal	Dried poultry protein	Chicken by‐product meal	Dried poultry protein
Main carbohydrate sources	Maize, wheat, soybean meal, brewers rice	Brewers rice, dried plain beet pulp	Brewers rice, wheat, barley	Barley, wheat gluten feed, maize gluten, tapioca
Metabolisable energy (kJ/100 g product)	1,556	1,705	1,446	1,442
Moisture (g/100 g)	8	9.5	9.5	9.5

	g/MJ ME	g/100 g DM	g/MJ ME	g/100 g DM	g/MJ ME	g/100 g DM	g/MJ ME	g/100 g DM
Crude fat	9.3	15.8	11.7	22.1	4.8	7.7	8.3	13.3
Crude protein	14.0	23.7	14.7	27.6	15.2	24.3	25.7	40.9
Crude fibre	1.1	1.9	0.9	1.8	1.2	1.9	4.4	7.1
Carbohydrates	31.6	53.5	21.7	40.9	36.8	58.8	20.7	33
Ash	3.1	5.2	4.0	7.6	4.6	7.3	3.6	5.7

^†^ When compared to the maintenance diet.

### 2.3 Monitoring

Every week, physical examinations of all dogs were carried out to monitor health status and detect early signs of gastrointestinal upset following dietary change.

Additionally, body weight and BCS were assessed every week to determine preservation of body weight and body condition during the trial and to increase or decrease the dietary quantity accordingly. Examinations and BCS assessments were performed by the same investigator. Dogs with severe gastrointestinal disease or weight change of more than 10% of their starting body weight were excluded from the trial.

### 2.4 Sample collection

Blood was sampled by jugular venipuncture and collected in serum tubes with clotting activator. After centrifugation, serum was collected and stored at −20°C until analysis to avoid multiple freezing and thaw cycles.

### 2.5 Assay validation and sample analysis

Precision was estimated by calculating the inter‐ and intra‐assay coefficients of variations (CV). Inter‐assay CV was determined by analysing two serum samples of lean dogs on five assays, performed on three different days. Intra‐assay CV was calculated by comparing the same two samples five times in one assay.

Serum leptin concentrations were measured with the use of commercial canine leptin sandwich ELISA kits (EZCL‐31K; Millipore) with a limit of detection of 0.21 ng/ml. Canine samples diluted parallel to the standard curve. The intra‐assay CV was 5.3%; the inter‐assay CV was 7.0%. For adiponectin, a previously validated human high sensitivity adiponectin ELISA kit (Human Adiponectin ELISA, High Sensitivity Kit; BioVendor–Laboratorni medicina) was used (Tvarijonaviciute, Martinez‐Subiela, & Ceron, 2010) with a limit of detection of 0.47 ng/ml. The canine samples diluted parallel to the standard curve. The intra‐assay CV was 8.0%; the inter‐assay CV was 11.0%. Analyses were performed according to the manufacturers' instructions. To limit the influence of inter‐assay variability, all samples of individual dogs were analysed on the same ELISA plate. Measurements were performed by a researcher that was blinded to the individual dogs and the diets.

### 2.6 Statistical analysis

Statistical analyses were performed by commercial software (IBM SPSS Statistics for Windows, version 25.0. IBM Corporation). Data were tested for normality using Kolmogorov–Smirnov tests and Q–Q plots. AUCs from/T to T_9_ (AUC_T−1–T9_) were calculated using the trapezoidal method as an estimation of the total release in a ten‐hour time span.

Differences in age and weight between groups were compared with an analysis of variance (ANOVA) with Bonferroni test as post hoc test for significant differences. Comparisons between mean body weight in each group and overall body weight before and after the trial were made with a paired *t* test. Differences in BCS between groups were compared with a Kruskal–Wallis test. Additional comparisons between BCS before and after the trial for each group and overall BCS were made with the Wilcoxon signed‐rank test with Bonferroni correction. Serum leptin and adiponectin concentrations did not comply to the assumptions of a repeated measures ANOVA, and the response of serum leptin and adiponectin concentrations to feeding during the control period at/T, T_3_, T_6_ and T_9_ were compared with Friedman tests and the Wilcoxon signed‐rank tests with Bonferroni correction.

Mixed model analyses for repeated measurements were performed on leptin peak concentrations (T_6_), leptin and adiponectin baseline concentrations (T_−1_) and AUC_T−1–T9_, with diet, gender, age, group and interactions as fixed factor. The animals were added as random factor to account for individual variations. Data of extreme outliers (>1.5 times interquartile range) were removed from this analysis. Models were built using step‐up selection with significant or trend factors and were compared using likelihood ratio tests. Pair‐wise comparisons with Bonferroni correction were made post hoc, and estimated marginal means were calculated. All data complied to the assumptions of mixed model analysis.

Serum leptin and adiponectin concentrations are presented as estimated marginal means ± *SE*. Other data are presented as means ± *SD*, unless stated otherwise. The level of significance was set at *p* < .05, results with 0.05 < *p *< .10 were considered a trend.

## 3 RESULTS

No dogs showed adverse events following dietary change, and no dogs were excluded from the trial. Gastrointestinal signs, such as diarrhoea or vomiting, were not observed. T_9_ samples of two dogs could not be collected during the maintenance period and were omitted from the post‐prandial effect analysis and the mixed model of the AUC_T−1–T9_ of leptin and adiponectin. Age (*p* = .16), body weight before (*p* = .43) and after (*p* = .19) the trial and BCS before (*p* = .32) and after (*p* = .32) the trial did not differ significantly between groups (Table 1). Body weight (*p* = .048) and BCS (*p* = .046) in group 2 reduced significantly at the end of the trial (*p* = .048), despite eating the adjusted amounts of food and without showing gastrointestinal signs, but all dogs remained within the 10% limit of their starting body weight.

### 3.1 Serum leptin concentrations

**Figure 1:**
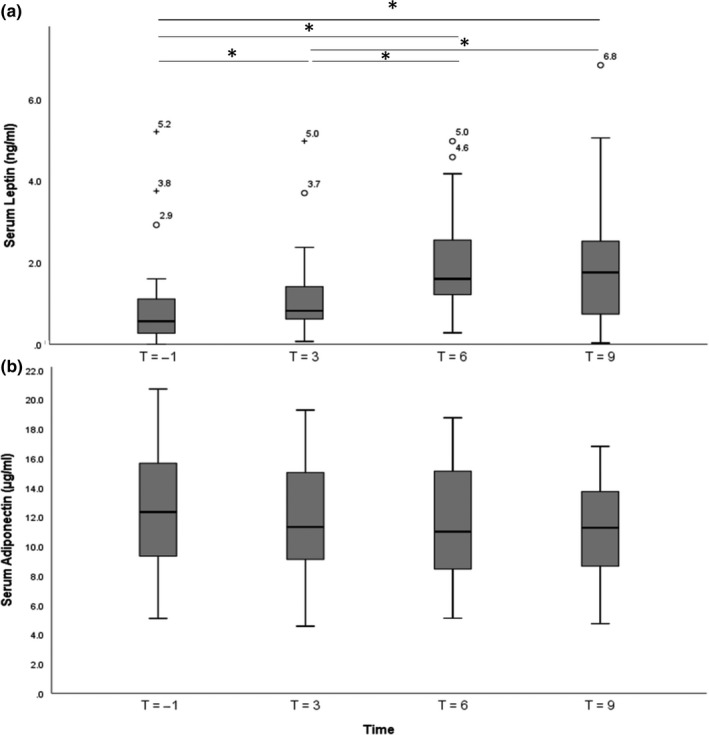
Box and whisker plots showing serum leptin (ng/ml) (a) and serum adiponectin (μg/ml) (b) concentrations at one hour before feeding and at three, six and nine hours after feeding the maintenance diet (*n* = 34). Outliers are presented as °, extreme outliers (>1.5 times the interquartile range) are presented as +. Significant differences (*p* < .05) between time points are presented as *

Twelve samples of 6 dogs fell under the detection limit of the leptin assay and were regarded as 0 ng/ml. Dietary intake increased serum leptin concentrations (*n* = 34, *p* < .001) (Figure 1). Post hoc analysis showed that/T differed from T_3_, T_6_ and T_9_, and T_3_ differed from T_6_ and T_9_. T_6_ and T_9_ did not differ, and T_6_ was regarded as the peak leptin concentration in further analyses.

The effects of diet on leptin concentrations are listed in Table 3. Five outliers were excluded from the analysis of baseline leptin concentrations, 3 from analysis of peak leptin concentrations and leptin AUC_T−1–T9_. Baseline leptin concentrations were not affected by diet (*p* = .273). Diet affected leptin peak (T_6_) concentrations (*p* = .002). The HP diet provided the lowest leptin peak concentrations (*p* = .004), and a lower total leptin release as measured by the AUC_T−1–T9_ (*p* = .01), when compared to the HCLP diet. In all groups, the diet that followed the HCLP diet, that is the HF diet in group 1, the HCMP diet in group 2 and the HP diet in group 3, produced lower leptin peak leptin concentrations (*p* < .001) and AUC_T−1–T9_ (*p* < .001), which was revealed as an interaction between diet and group. No other factors or interactions altered leptin concentrations and were omitted from the final model.

**Table 3:** Estimated marginal means obtained by the mixed model of leptin baseline and peak concentrations (ng/ml) (*n* = 36) and AUC_T−1–T9_ (ng m^−1^ 10 hr^−1^) (*n* = 34) for each diet

Diet	Baseline leptin concentration (ng/ml)		Peak leptin concentration (ng/ml)		Leptin AUC_T__−__1_ *_–_*_T9_ (ng ml^−1^ 10 hr^−1^)	
	Mean ± *SEM*	CI	Mean ± *SEM*	CI	Mean ± *SEM*	CI
Maintenance diet	0.9 ± 0.13	0.6–1.1	1.9 ± 0.15	1.6–2.2	13.7 ± 1.13	11.4–15.9
High‐protein diet	0.7 ± 0.13	0.5–1.0	1.5* ± 0.15	1.2–1.8	11.4* ± 1.11	9.2–13.6
High carbohydrate medium Protein diet	0.7 ± 0.13	0.4–0.9	1.8 ± 0.15	1.5–2.1	12.4 ± 1.10	10.2–14.7
High‐fat diet	0.8 ± 0.13	0.6–1.1	1.9 ± 0.15	1.6–2.2	13.7 ± 1.11	11.5–15.9

Abbreviations: AUC, Area under the curve from/T to T_9_; CI, 95% Confidence Interval; *SEM*, Standard Error of the Mean.

* Significant difference when compared to the maintenance diet (*p* < .05).

### 3.2 Serum adiponectin concentrations

All samples were above the detection limit of the adiponectin assay. In contrast to leptin, feeding did not change serum adiponectin concentrations (*n* = 34, *p* = .056) (Figure 1b). Consequently, peak adiponectin concentrations could not be determined. Two outliers were removed from the analysis of baseline adiponectin concentrations, and 3 outliers were removed from analysis of adiponectin AUC_T−1–T9_.

The effects of the diet on adiponectin concentrations are summarised in Table 4. Diet significantly influenced baseline serum adiponectin concentrations (*p* < .001), with both the HF (*p* = .018) and the HCMP diet (*p* < .001) causing lower concentrations than the HCLP diet. Adiponectin AUC_T−1–T9_ was similarly influenced by dietary composition (*p* < .001), showing lower adiponectin release with the HP (*p* = .039), HF (*p* = .05) and HCMP diet (*p* < .001), when compared to the HCLP diet.

**Table 4:** Estimated marginal means of adiponectin baseline concentrations (μg/ml) (*n* = 36) and AUC_T−1–T9_ (μg ml^−1 ^10 hr^−1^) (*n* = 34) for each diet and gender as obtained by the mixed model

	Adiponectin Baseline Concentration (μg/ml)		Adiponectin AUC_T__−__1_ *_–_*_T9_ (μg/ml)	
	Mean ± *SEM*	CI	Mean ± *SEM*	CI
Diet†				
Maintenance diet	12.6 ± 0.63	11.4–13.9	122.2 ± 5.68	110.7–123.4
High‐protein diet	11.8 ± 0.63	10.6–13.1	114.2* ± 5.68	102.7–125.7
High carbohydrate medium protein diet	10.7* ± 0.62	9.4–12.0	106.7* ± 5.68	95.2–118.1
High‐fat diet	11.4* ± 0.62	10.2–12.7	111.9* ± 5.70	100.5–123.4
Gender†				
Male	12.9 ± 0.93	11.0–14.7	126.7 ± 8.81	108.8–144.6
Female	10.4** ± 0.65	9.1–11.8	100.8** ± 6.19	88.2–113.4

Abbreviations: AUC, Area under the curve from/T to T_9_; CI, 95% Confidence Interval; *SEM*, Standard Error of the Mean.

* Significant difference when compared to the maintenance diet (*p* < .05).

** Significant difference when compared to male dogs (*p* < .05).

^†^ Evaluated at age: 4.7 years.

In addition, male dogs had significantly higher baseline adiponectin concentrations (*p* = .041) and AUC_T−1–T9_ (*p* = .023) than female dogs (Table 4). Adiponectin AUC_T−1–T9_ was also influenced by age (*p* = .082), which increased with 2.86 μg ml^−1^ 10 hr^−1^ per year of age. No other factors or interactions influenced adiponectin concentrations.

## 4 DISCUSSION

This study identified variations in circulating leptin and adiponectin concentrations in response to different dietary macronutrient composition in overweight dogs, without combining diet with subsequent weight loss (Mitsuhashi et al., 2010) or neutering (Kawauchi et al., 2017). By preventing weight gain or weight loss, and using each dog as its own control, the effects of body weight and body composition are negligible. Manipulation of the circulating concentrations of these hormones could improve immune‐metabolic health in overweight individuals (Cortese et al., 2019), even before achieving weight loss. The HP diet decreased post‐prandial release of leptin, while causing the lowest increase of adiponectin concentrations compared with the other test diets.

The current findings contribute to previous work on the effects of dietary macronutrient composition on glucose metabolism‐ and satiety‐related hormones in dogs (Kawauchi et al., 2017; Schauf et al., 2016, 2018). Previously, lower post‐prandial increases of cholecystokinin (CCK) and peptide YY (PYY) with a HF diet (crude protein (CP): 30.7 g/100 g dry matter (DM); crude fat (CF): 21.3 g/100 g DM; and carbohydrates: 32.2 g/100 g DM) when compared to a HC diet (CP: 26.9 g/100 g DM; CF: 10.5 g/100 g DM; carbohydrates: 46.6 g/100 g DM) were found. Another study increased basal concentrations of glucagon‐like peptide 1 (GLP‐1) with a HF diet (CP: 30.0 g/100 g DM; CF: 21.4 g/100 g DM; and carbohydrates: 39.4 g/100 g DM) when compared to a HC diet (CP: 25.9 g/100 g DM; CF: 9.9 g/100 g DM; and carbohydrates: 54.9 g/100 g DM) (Schauf et al., 2018). Additionally, a comparison of a diet with dietary protein at recommended maintenance level (CP: 21.5 g/100 g DM; CF: 31.0 g/100 g; and carbohydrates: 49.0 g/100 g DM) and a HP diet (CP: 33.8 g/100 g DM; HF: 29.4 g/100 g DM; and carbohydrates: 38.6 g/ 100 g DM) did not cause alterations in basal concentrations of glucagon, leptin, insulin and insulin‐like growth factor 1 (IGF‐1) (Kawauchi et al., 2017). In the present study, the effect of the HP diet on the post‐prandial leptin suggests a role of dietary protein in the regulation of long‐term satiety (Kawauchi et al., 2017; Orr & Davy, 2005).

Release of leptin and adiponectin from adipocytes is closely related to glucose metabolism and the balance between pro‐ and anti‐inflammatory cytokines. Leptin is released when circulating insulin concentrations and pro‐inflammatory cytokines increase in humans, the latter also inducing leptin resistance (Park & Ahima, 2015; Sáinz, Barrenetxe, Moreno‐Aliaga, & Martinez, 2015; Seufert, 2004). Lower insulinemic responses and decreases in inflammatory cytokines, as observed in dogs and obese humans when using HP diets (Amini, Maghsoudi, Feizi, Ghiasvand, & Askari, 2016; André et al., 2017), could thus decrease the post‐prandial leptin release. The observed effects could also be associated with the relative lack of carbohydrates and dietary fat in the HP diet, as these macronutrients have been associated with leptin resistance (Giugliano, Ceriello, & Esposito, 2006; Koch et al., 2014).

Circulating adiponectin concentrations, on the other hand, are inversely related to insulin concentrations (Gayet, Leray, Saito, Siliart, & Nguyen, 2007; Pellmé et al., 2003), but were not increased with the HP diet. Previously reported increases in circulating adiponectin concentrations after weight loss with HP diets could thus be the result of weight loss, rather than alterations in macronutrient composition (André et al., 2017; Ata et al., 2010; Tvarijonaviciute, Tecles, Martinez‐Subiela, & Cerón, 2012). It is, however, possible that the maintenance diet already provided optimal adiponectin concentrations, as feeding HC diets to cats increases circulating adiponectin concentrations (Tan et al., 2011). As adiponectin has a strong anti‐inflammatory effect (Radin et al., 2009), it is possible that the test diets exacerbate low‐grade inflammation. However, overweight individuals have lower adiponectin concentrations than lean individuals, and the biologic effects of further decrease are unknown (André et al., 2017; Bastien et al., 2014; Ishioka et al., 2006; Tvarijonaviciute, Tecles, et al., 2012).

The post‐prandial release of leptin was decreased with the HP diet, despite the fact that most overweight individuals have hyperleptinemia sequential to leptin resistance (Cortese et al., 2019; Park et al., 2014). As dietary intake leads to prolonged periods of high leptin concentrations, with a maximal concentration around six to nine hours after feeding (Ishioka et al., 2005), lower post‐prandial leptin release could alleviate signs of hyperleptinemia for a prolonged period, improving post‐prandial satiety, insulin sensitivity and immune‐metabolic health (Cortese et al., 2019; Radin et al., 2009; Tvarijonaviciute, Tecles, et al., 2012).

Leptin concentrations in the present study were considerably lower than a previous report in dogs that used the same assay (Park et al., 2014). This discrepancy might originate from the use of solely intact dogs, as opposed to the several neutered dogs in the study of Park et al. (2014), although it was previously suggested neutering status does not affect circulating leptin concentrations (Ishioka et al., 2007). In accordance with preceding studies, baseline leptin concentrations were not affected by diet (Adolphe et al., 2015; Kawauchi et al., 2017), which is likely to be the result of the lack of overall change in body composition during the present study (Ishioka et al., 2007; Park & Ahima, 2015). Leptin concentrations were also not influenced by age and gender, as was previously shown by Ishioka et al. (2007), which makes fasting leptin concentrations a reliable marker of changes in fat mass without being confounded by age, gender and diet when the time of feeding is stated (Ishioka et al., 2005).

From a physiologic perspective, it may be expected that adiponectin concentrations decrease post‐prandially to limit its effects on energy uptake and expenditure (Lee & Shao, 2014). Although in the present study, numerically, the highest concentrations of adiponectin were found before dietary intake and the values decreased after food intake, these differences were not statistically significant, as was also reported by Tvarijonaviciute, Cerón, and Tecles (2012). The use of overweight dogs in this study, in which adiponectin release is already decreased (Ishioka et al., 2006; Tvarijonaviciute et al., 2010), might account for this lack of response to food intake.

In the present study, the concentrations of adiponectin measured with a human adiponectin assay were comparable to a study that validated this assay (Tvarijonaviciute et al., 2010). In contrast to what was observed by Verkest et al. (2011), we found a significant gender effect with regard to adiponectin concentrations, with male dogs having higher adiponectin concentrations than female dogs. This sex dimorphism disagrees with studies in humans, where testosterone inhibits the release of adiponectin from adipocytes (Xu et al., 2005). Other endocrine influences might contribute to this dimorphism in dogs, as was suggested previously (Verkest et al., 2011).

A limitation of using commercial diets is the variation in ingredients in each diet, which could have interfered with the results. Considering the effects of different sources of carbohydrates (Carciofi et al., 2008) and proteins (Nuttal, Gannon, Wald, & Ahmed, 1985) on the post‐prandial insulin response and sensitivity, it is expected that micronutrient composition also influences the regulation of circulating leptin and adiponectin concentration. Future studies, preferably with experimental diets, are needed to accurately assess these effects on adipokine release. Additionally, no golden standard of a “normal” diet exists in veterinary nutrition. In the present study a maintenance diet, high in carbohydrates and low in proteins in comparison with our test diets, was considered closest to a control diet. To exclude the presence of a carry‐over effect due to composition, which could explain the interaction that was found between group and diet for leptin concentrations, using a Latin square design with complete randomisation and inclusion of wash‐out periods between diets would have been preferred.

## 5 CONCLUSIONS

This study is the first to show the beneficial effects of a HP diet on leptin concentrations, while causing minimal decrease in adiponectin concentrations in overweight dogs without combining dietary change with weight loss. Lower leptin concentrations suggest improved sensitivity to the hormone, thus increasing post‐prandial satiety, improving insulin sensitivity and lowering obesity‐related inflammation (Cortese et al., 2019; Sáinz et al., 2015). The fact that only post‐prandial concentrations of leptin could be altered, might suggest that its baseline levels are foremost dependent on the fat mass of an individual rather than influenced by dietary macronutrients (Ishioka et al., 2007; Park et al., 2014). Adiponectin concentrations could not be increased, but the HF diet, and the HCMP diet decreased baseline adiponectin concentrations and the HP, HF and HCMP diet decreased post‐prandial adiponectin release, which could decrease insulin sensitivity and increase obesity‐related inflammation (André et al., 2017; Bastien et al., 2014; Tvarijonaviciute, Tecles, et al., 2012). To this end, a HP diet might improve immune‐metabolic health by decreasing leptin concentrations even before weight loss is achieved, without decreasing baseline adiponectin concentrations and providing minimal decrease in adiponectin concentrations.

## CONFLICT OF INTEREST

The authors declare no conflict of interest. The diets were kindly provided by Royal Canin, but this company was not involved in study design nor the analysis of the results.

## ANIMAL WELFARE STATEMENT

The authors confirm that the ethical policies of the journal, as noted on the journal's author guidelines page, have been adhered to and the appropriate ethical review committee approval has been received. The authors confirm that they have followed EU standards for the protection of animals used for scientific purposes. The protocol and study design were approved by the Animal Ethics Committee at Utrecht University (registered under number AVD1080020184847) and the Royal Canin Ethics Committee.

## References

[jpn13285-bib-0001] Adolphe, J. L. , Drew, M. D. , Silver, T. I. , Fouhse, J. , Childs, H. , & Weber, L. P. (2015). Effect of an extruded pea or rice diet on postprandial insulin and cardiovascular responses in dogs. Journal of Animal Physiology and Animal Nutrition, 99, 767–776. 10.1111/jpn.12275 25475789

[jpn13285-bib-0002] Amini, P. , Maghsoudi, Z. , Feizi, A. , Ghiasvand, R. , & Askari, G. (2016). Effects of high protein and balanced diets on lipid profiles and inflammation biomarkers in obese and overweight women at aerobic clubs: A randomized clinical trial. International Journal of Preventive Medicine, 7, 110 10.4103/2008-7802.190608 27833724 PMC5036282

[jpn13285-bib-0003] André, A. , Leriche, I. , Chaix, G. , Thorin, C. , Burger, M. , & Nguyen, P. (2017). Recovery of insulin sensitivity and optimal body composition after rapid weight loss in obese dogs fed a high‐protein medium‐carbohydrate diet. Journal of Animal Physiology and Animal Nutrition, 101, 21–30. 10.1111/jpn.12744 28627053

[jpn13285-bib-0004] Ata, S. M. , Vaishnav, U. , Puglisi, M. , Lofgren, I. E. , Wood, R. J. , Volek, J. S. , & Fernandez, M. L. (2010). Macronutrient composition and increased physical activity modulate plasma adipokines and appetite hormones during a weight loss intervention. Journal of Women's Health, 19, 139–145. 10.1089/jwh.2009.1472 20088670

[jpn13285-bib-0005] Bastien, B. C. , Patil, A. , & Satyaraj, E. . (2014). The impact of weight loss on circulating cytokines in Beagle dogs. Veterinary Immunology and Immunopathology, 163, 174–182. 10.1016/j.vetimm.2014.12.003 25576490

[jpn13285-bib-0006] Carciofi, A. C. , Takakura, F. S. , de‐Oliveira, L. D. , Teshima, E. , Jeremias, J. T. , Brunetto, M. A. , & Prada, F. (2008). Effects of six carbohydrate sources on dog diet digestibility and post‐prandial glucose and insulin response. Journal of Animal Physiology and Animal Nutrition, 92, 326–336. 10.1111/j.1439-0396.2007.00794.x 18477314

[jpn13285-bib-0007] Cortese, L. , Terrazzano, G. , & Pelagalli, A. (2019). Leptin and immunological profile in obesity and its associated diseases in dogs. International Journal of Molecular Sciences, 20, E2392 10.3390/ijms20102392 31091785 PMC6566566

[jpn13285-bib-0008] Gayet, C. , Leray, V. , Saito, M. , Siliart, B. , & Nguyen, P. (2007). The effects of obesity‐associated insulin resistance on mRNA expression of peroxisome proliferator‐activated receptor gamma target genes, in dogs. The British Journal of Nutrition, 98, 497–503. 17475082 10.1017/S000711450772514X

[jpn13285-bib-0009] German, A. J. , Hervera, M. , Hunter, L. , Holden, S. L. , Morris, P. J. , Biourge, V. , & Trayhurn, P. (2009). Improvement in insulin resistance and reduction in plasma inflammatory adipokines after weight loss in obese dogs. Domestic Animal Endocrinology, 37, 214–226. 10.1016/j.domaniend.2009.07.001 19674864

[jpn13285-bib-0010] German, A. J. , Holden, S. L. , Bissot, T. , Hackett, R. M. , & Biourge, V. (2007). Dietary energy restriction and successful weight loss in obese client‐owned dogs. Journal of Veterinary Internal Medicine, 21, 1174–1180. 10.1111/j.1939-1676.2007.tb01934.x 18196722

[jpn13285-bib-0011] Giugliano, D. , Ceriello, A. , & Esposito, K. (2006). The effects of diet on inflammation: Emphasis on the metabolic syndrome. Journal of the American College of Cardiology, 48, 677–685. 10.1016/j.jacc.2006.03.052 16904534

[jpn13285-bib-0012] Ishioka, K. , Hatai, H. , Komabayashi, K. , Soliman, M. M. , Shibata, H. , Honjoh, T. , … Saito, M. (2005). Diurnal variation of serum leptin in dogs: Effects of fasting and re‐feeding. Veterinary Journal, 169, 85–90. 10.1016/j.tvjl.2004.01.003 15683767

[jpn13285-bib-0013] Ishioka, K. , Hosoya, K. , Kitagawa, H. , Shibata, H. , Honjoh, T. , Kimura, K. , & Saito, M. (2007). Plasma leptin concentration in dogs: Effects of body condition score, age, gender and breeds. Research in Veterinary Medicine, 82, 11–15. 10.1016/j.rvsc.2006.06.002 16919691

[jpn13285-bib-0014] Ishioka, K. , Omachi, A. , Sagawa, M. , Shibata, H. , Honjoh, T. , Kimura, K. , & Saito, M. (2006). Canine adiponectin: cDNA structure, mRNA expression in adipose tissues and reduced plasma levels in obesity. Research in Veterinary Medicine, 80, 127–132. 10.1016/j.rvsc.2005.05.011 16051287

[jpn13285-bib-0015] Jeusette, I. C. , Detilleux, J. , Shibata, H. , Saito, M. , Honjoh, T. , Delobel, A. , … Diez, M. (2005). Effects of chronic obesity and weight loss on plasma ghrelin and leptin concentrations in dogs. Research in Veterinary Medicine, 79, 169–175. 10.1016/j.rvsc.2004.11.012 15924935

[jpn13285-bib-0016] Kawauchi, I. M. , Jeremias, J. T. , Takeara, P. , de Souza, D. F. , Balieriro, J. C. C. , Pfrimer, K. , … Pontieri, C. F. F. (2017). Effect of dietary protein intake on the body composition and metabolic parameters of neutered dogs. Journal of Nutritional Science, 6, e40 10.1017/jns.2017.41 29152244 PMC5672309

[jpn13285-bib-0017] Kealy, R. D. , Lawler, D. F. , Ballam, J. M. , Lust, G. , Biery, D. N. , Smith, G. K. , & Mantz, S. L. (2000). Evaluation of the effect of limited food consumption on radiographic evidence of osteoarthritis in dogs. Journal of the American Veterinary Medical Association, 217, 1678–1680. 10.2460/javma.2000.2017.1678 11110459

[jpn13285-bib-0018] Kealy, R. D. , Lawler, D. F. , Ballam, J. M. , Mantz, S. L. , Biery, D. N. , Greeley, E. H. , … Stowe, H. D. (2002). Effects of diet restriction on life span and age‐related changes in dogs. Journal of the American Veterinary Medical Association, 220, 1315–1320. 10.2460/javma.2002.220.1315 11991408

[jpn13285-bib-0019] Koch, C. E. , Lowe, C. , Pretz, D. , Steger, J. , Williams, L. M. , & Tups, A. (2014). High‐fat diet induces leptin resistance in leptin‐deficient mice. Journal of Neuroendocrinology, 26, 58–67. 10.1111/jne.12131 24382295

[jpn13285-bib-0020] LaFlamme, D. P. (1997). Development and validation of a body condition score system for dogs. Canine Practice, 22, 10–15.

[jpn13285-bib-0021] Lee, B. , & Shao, J. (2014). Adiponectin and energy homeostasis. Reviews in Endocrine & Metabolic Disorders, 15, 149–156. 10.1007/s11154-013-9283-3 24170312 PMC4006341

[jpn13285-bib-0022] Minokoshi, Y. , Kim, Y. B. , Peroni, O. D. , Fryer, L. G. , Müller, C. , Carling, D. , & Kahn, B. B. (2002). Leptin stimulates fatty‐acid oxidation by activating AMP‐activated protein kinase. Nature, 415, 339–343. 10.1038/415339a 11797013

[jpn13285-bib-0023] Mitsuhashi, Y. , Nagaoka, D. , Ishioka, K. , Bigley, K. E. , Okawa, M. , Otsuji, K. , & Bauer, J. E. (2010). Postprandial lipid‐related metabolites are altered in dogs fed dietary diacylglycerol and low glycemic index starch during weight loss. The Journal of Nutrition, 140, 1815–1823. 10.3945/jn.110.122887 20739444

[jpn13285-bib-0024] Nuttall, F. Q. , Gannon, M. C. , Wald, J. L. , & Ahmed, M. (1985). Plasma glucose and insulin profiles in normal subjects ingesting diets of varying carbohydrate, fat, and protein content. Journal of the American College of Nutrition, 4, 437–450. 10.1080/07315724.1985.10720086 3900180

[jpn13285-bib-0025] Orr, J. , & Davy, B. (2005). Dietary influences on peripheral hormones regulating energy intake: Potential applications for weight management. Journal of the American Dietetic Association, 105, 1115–1127. 10.1016/j.jada.2005.04.005 15983531

[jpn13285-bib-0026] Park, H. K. , & Ahima, R. S. (2015). Physiology of leptin: Energy homeostasis, neuroendocrine function and metabolism. Metabolism: Clinical and Experimental, 64, 24–34. 10.1016/j.metabol.2014.08.004 25199978 PMC4267898

[jpn13285-bib-0027] Park, H. J. , Lee, S. E. , Oh, J. H. , Seo, K. W. , & Song, K. H. (2014). Leptin, adiponectin and serotonin levels in lean and obese dogs. BMC Veterinary Research, 10, 113 10.1186/1746-6148-10-113 24886049 PMC4030042

[jpn13285-bib-0028] Pellmé, F. , Smith, U. , Funahashi, T. , Matsuzawa, Y. , Brekke, H. , Wiklund, O. , … Jansson, P. A. (2003). Circulating adiponectin levels are reduced in nonobese but insulin‐resistant first‐degree relatives of type 2 diabetic patients. Diabetes, 52, 1182–1186. 10.2337/diabetes.52.5.1182 12716750

[jpn13285-bib-0029] Radin, M. J. , Sharkey, L. C. , & Holycross, B. J. (2009). Adipokines: A review of biological and analytical principles and an update in dogs, cats, and horses. Veterinary Clinical Pathology, 38, 136–156. 10.1111/j01939-165X.2009.00133.x 19392760

[jpn13285-bib-0030] Sáinz, N. , Barrenetxe, J. , Moreno‐Aliaga, M. J. , & Martinez, J. A. (2015). Leptin resistance and diet‐induced obesity: Central and peripheral actions of obesity. Metabolism: Clinical and Experimental, 64, 35–46. 10.1016/j.metabol.2014.10.015 25497342

[jpn13285-bib-0031] Schauf, S. , Salas‐Mani, A. , Torre, C. , Bosch, G. , Swarts, H. , & Castrillo, C. (2016). Effects of sterilization and of dietary fat and carbohydrate content on food intake, activity level, and blood satiety‐related hormones in female dogs. Journal of Animal Science, 94, 4239–4250. 10.2527/jas.2015-0109 27898845

[jpn13285-bib-0032] Schauf, S. , Salas‐Mani, A. , Torre, C. , Jimenez, E. , Latorre, M. A. , & Castrillo, C. (2018). Effect of feeding a high‐carbohydrate or a high‐fat diet on subsequent food intake and blood concentration of satiety‐related hormones in dogs. Journal of Animal Physiology and Animal Nutrition, 102, e21–e29. 10.1111/jpn.12696 28447374

[jpn13285-bib-0033] Seufert, J. (2004). Leptin effects on pancreatic beta‐cell gene expression and function. Diabetes, 53, S152–S158. 14749281 10.2337/diabetes.53.2007.s152

[jpn13285-bib-0034] Tan, H. Y. , Rand, J. S. , Morton, J. M. , Fleeman, L. M. , Armstrong, P. J. , Coradinin, M. , … Whitehead, J. P. (2011). Adiponectin profiles are affected by chronic and acute changes in carbohydrate intake in healthy cats. General and Comparative Endocrinology, 172, 468–474. 10.1016/j.ygcen.2011.04.012 21530529

[jpn13285-bib-0035] Tvarijonaviciute, A. , Cerón, J. J. , & Tecles, F. (2012). Serum adiponectin concentrations in dogs_–_abscence of diurnal variation and lack of effect of feeding and methylprednisolone administration. Acta Veterinaria Hungarica, 60, 489–500. 10.1556/AVet2012.043 23160031

[jpn13285-bib-0036] Tvarijonaviciute, A. , Martínez‐Subiela, S. , & Ceron, J. J. (2010). Validation of 2 commercially available enzyme‐linked immunosorbent assays for adiponectin determination in canine serum samples. Canadian Journal of Veterinary Research, 74, 279–285. 21197228 PMC2949341

[jpn13285-bib-0037] Tvarijonaviciute, A. , Tecles, F. , Martinez‐Subiela, S. , & Cerón, J. J. (2012). Effect of weight loss on inflammatory biomarkers in obese dogs. Veterinary Journal, 193, 570–572. 10.1016/j.tvjl.2012.02.015 22464400

[jpn13285-bib-0038] Verkest, K. R. , Rose, F. J. , Fleeman, L. M. , Rand, J. S. , Morton, J. M. , Richards, A. A. , … Whitehead, J. P. (2011). Adiposity and adiponectin in dogs: Investigation of causes of discrepant results between two studies. Domestic Animal Endocrinology, 41, 35–41. 10.1013/j.domaniend.2011.03.004 21645805

[jpn13285-bib-0039] Vetter, M. L. , Wade, A. , Womble, L. G. , Dalton‐Bakes, C. , Wadden, T. A. , & Igbal, N. (2010). Effect of a low‐carbohydrate diet versus a low‐fat, calorie‐restriction diet on adipokine levels in obese, diabetic participants. Diabetes, Metabolic Syndrome and Obesity: Targets and Therapy, 3, 357–361. 10.2147/DMSOTT.S13966 PMC3047958 21437105

[jpn13285-bib-0040] Xu, A. , Chan, K. W. , Hoo, R. L. , Wang, Y. , Tan, K. C. , Zhang, J. , … Lam, K. S. (2005). Testosterone selectively reduces the high molecular weight form of adiponectin by inhibiting its secretion from adipocytes. Journal of Biological Chemistry, 280, 18073–18080. 10.1074/jbc.M414231200 15760892

